# Loss of DDX24 inhibits lung cancer progression by stimulating IKBKG splicing-mediated autophagy

**DOI:** 10.7150/thno.102425

**Published:** 2025-01-02

**Authors:** Siwen Sun, Xiaomeng Jing, Guangquan Tong, Chaoqun Chen, Shuaijun Xie, Chong Wang, Dan Chen, Jinyao Zhao, Yangfan Qi, Wenjing Zhang, Congcong Liu, Ge Zhang, Jinrui Zhang, Bing Sun, Yang Wang, Yuesheng Lv

**Affiliations:** 1 Department of Oncology & Sino-US Research Center for Cancer Translational Medicine, the Second Affiliated Hospital of Dalian Medical University, Dalian Medical University, Dalian 116023, China.; 2 Sino-US Research Center for Cancer Translational Medicine of the Second Affiliated Hospital of Dalian Medical University & Institute of Cancer Stem Cell, Dalian Medical University, Dalian 116023, China.; 3 Department of Urology, The First Affiliated Hospital of Jinzhou Medical University, Jinzhou 121001, China.; 4 Department of Pathology, the First Affiliated Hospital of Dalian Medical University, Dalian Medical University, Dalian 116011, China.; 5 Institute of Cancer Stem Cell, Dalian Medical University, Dalian 116044, China.; 6 Department of Immunology, College of Basic Medical Sciences, Dalian Medical University, Dalian 116044, China.; 7 Department of Thoracic Surgery, the First Affiliated Hospital of Dalian Medical University, Dalian Medical University, Dalian 116011, China.

**Keywords:** DDX24, alternative splicing, IKBKG, autophagy, lung cancer

## Abstract

**Rationale:** Lung cancer remains a major global health burden with limited therapeutic options. Alternative splicing, a critical post-transcriptional process, contributes to lung cancer progression through autophagy, although the underlying mechanisms remain largely unexplored. This study aims to elucidate the role of DDX24 as a splicing factor that contributes to lung cancer progression via autophagy.

**Methods:** To establish the link between DDX24 and lung cancer progression, we performed colony formation assays, growth curve analyses, and xenograft tumor models in nude mice. Mass spectrometry and RNA sequencing were employed to investigate the involvement of DDX24 in alternative splicing, with a specific focus on the splicing of IKBKG. The mechanisms by which DDX24 regulates autophagy were further explored using co-immunoprecipitation and luciferase reporter assays.

**Results:** The splicing factor DDX24 is significantly elevated in lung cancer tissues. Loss of DDX24 suppresses lung cancer growth by promoting autophagy. We identified DDX24 as a splicing factor that plays critical roles in the regulation of alternative splicing. Mechanistically, DDX24 regulates the alternative splicing of autophagy-related genes, including IKBKG. We demonstrate that DDX24 directly binds to IKBKG pre-mRNA, whereas DDX24 ablation stimulates the generation of the long splicing isoform of IKBKG, thereby promoting autophagy through activating of the NF-kB signaling pathway and the transcription of the BECN1 gene. Functional rescue experiments confirm that the long IKBKG isoform-mediated autophagy confers the anti-tumor effects of DDX24 depletion. In addition, IKBKG-L is positively associated with improved survival in lung cancer patients.

**Conclusions:** This study uncovers a novel regulatory axis involving DDX24, IKBKG splicing, and autophagy in lung cancer. Our findings suggest that targeting DDX24 may represent a promising therapeutic strategy for lung cancer treatment, offering new insights into the molecular underpinnings of this disease.

## Introduction

Lung cancer represents a significant global health burden, ranking first in both incidence and mortality rates. An estimated 2.2 million new cases and 1.8 million deaths were attributed to lung cancer in 2020 1. Notably, it is the most lethal cancer in men and the second most lethal in women, trailing only breast cancer 2. Histologically, lung cancer can be categorized into two major subtypes: non-small cell lung cancer (NSCLC) and small cell lung cancer (SCLC). NSCLC can be further subclassified based on histopathological features into adenocarcinoma (LAD), squamous cell carcinoma (LSCC), and large cell carcinoma (LLCC) 3.

While pathological classification has provided a framework for understanding of lung cancer, next-generation genomic sequencing has unveiled substantial intratumoral heterogeneity within individual pathological subtypes. This has led to a paradigm shift in lung cancer management, with increasing emphasis on biopsy and tumor sequencing to guide therapeutic decisions. For example, a subset of NSCLC patients harboring actionable mutations like EGFR, ALK, ROS1, BRAF, or MET can benefit from first-line treatment with targeted small-molecule inhibitors 4. However, the high degree of intratumoral heterogeneity necessitates alternative therapeutic strategies, such as cytotoxic chemotherapy, for patients lacking identifiable driver mutations 5. Therefore, elucidating the underlying mechanisms of lung cancer development remains crucial for improving therapeutic outcomes. Identifying additional driver mutations holds significant promise for advancing personalized medicine approaches in lung cancer.

Autophagy, a highly conserved intracellular degradation system in eukaryotes, serves a critical role in maintaining cellular homeostasis under starvation conditions 6. By targeting redundant proteins and dysfunctional organelles for degradation, autophagy provides essential nutrients and energy to sustain basal cellular activities, particularly during starvation. The initiation of autophagy is primarily regulated by two signaling pathways: mTORC1 and AMPK pathways. These pathways respond to extracellular cues like nutrient deprivation and energy depletion, leading to the activation of the autophagic process via modulation of the ULK1 complex. Subsequently, the Beclin1-PIK3C3 complex facilitates the formation of phosphatidylinositol-3-phosphate (PI3P) on autophagic membranes. PI3P serves as a docking site for proteins crucial for autophagosome initiation, thereby triggering the autophagic cascade.

Following autophagosome formation, lysosomes fusion generates autolysosomes. Within these compartments, lysosomal proteases degrade autophagic substrates, enabling the recycling of cellular components for reuse. Autophagy is intricately linked to various pathologies, including neurodegenerative disorders and cancer 7. While its primary function involves cellular adaptation to stress and maintenance of homeostasis, autophagy can also contribute to cell death under certain circumstances 8-10. Notably, several oncogenes, such as AKT, PI3K, Bcl-1, and mutated P53, can suppress autophagy. Conversely, prolonged autophagic activity can culminate in autophagic cell death 11,12.

Autophagy, a cellular stress response mechanism, exhibits a complex relationship with tumor progression. While activation of autophagic cell death has been observed during treatment with various anti-tumor agents, such as tamoxifen-induced autophagic death in breast cancer cells 13, and temozolomide-induced autophagic death in glioma 14. Other studies suggest autophagy can promote tumor cell resistance to chemotherapeutics. Unraveling the regulatory mechanisms governing autophagy and its multifaceted role in tumor development is crucial for optimizing the efficacy of chemotherapy and developing novel therapeutic strategies for cancer.

Alternative splicing (AS) is a fundamental process that governs mRNA and protein diversity by enabling a single pre-mRNA to generate different mature mRNAs through various exon inclusion/exclusion patterns 15. In addition, AS is tightly controlled during development and across distinct tissues 16,17. Importantly, dysregulation of AS plays crucial roles in the pathogenesis of various diseases, including cancer. Aberrant AS can manifest through two primary mechanisms: mutations within cis-acting regulatory elements on pre-mRNAs and altered expression of splicing factors. For instance, in lung cancer, frequent mutations near the 5' and 3' splice sites of MET lead to exon 14 skipping, disrupting METΔ14 binding to CBL, thereby promoting MET protein stability and ultimately contributing to tumorigenesis 18, 19. In addition, downregulated QKI is positively correlated with lung cancer progression and negatively associated with patient survival 20. Recent studies demonstrate QKI's role in regulating numerous lung cancer-related splicing events 21, including exon 11 skipping in NUMB 20, 21, and upregulation of the ED-B FN1 splice variant 22. Notably, alternative splicing can generate functionally distinct, even opposing, isoforms from the same gene. For example, Bcl-X can produce the anti-apoptotic long splicing isoform Bcl-XL, as well as the pro-apoptotic short isoform Bcl-XS, with RBM4 and SRSF1 modulating Bcl-X splicing, thereby influencing lung cancer development 23.

Despite the established role of aberrant AS in lung cancer development and progression, relatively few therapeutic targets addressing these splicing anomalies have been developed compared to the expanding landscape of lung cancer therapies. This highlights the urgent need to further explore AS as a potential source of novel and efficacious targets for improved lung cancer treatment.

Previous studies have implicated the DEAD-box RNA helicase DDX24 in vascular malformations 24. Additionally, DDX24 has been shown to interact with FADD and RIP1, influencing immune regulation and IRF7 activity 25. Furthermore, DDX24 can bind to P300, suppressing its acetyltransferase activity on p53 and consequently inhibiting p53 transcriptional activity 26. Notably, deregulated expression of DEAD-box RNA helicase family members is frequently observed in tumor tissues, suggesting their potential involvement in tumorigenesis 27-29. However, the specific role of DDX24 in lung cancer progression remains largely unknown and warrants further investigation.

IKBKG, also known as NEMO, serves as a key subunit within the IKK core complex. It plays a pivotal role in facilitating the dissociation of the inhibitory NF-kB complex, culminating in the degradation of the inhibitory protein. This process is essential for the subsequent activation of the NF-kB signaling cascade 30-32. Studies have reported that the interaction between IKBKG and TANK is critical for the activation of the NF-kB signaling pathway 33-34.

In this study, we found that DDX24 depletion significantly impaired the proliferative capacity of lung cancer cells. Mechanistically, we identified DDX24 as a regulator of autophagy-related gene splicing, including IKBKG. Further investigation revealed that DDX24 knockdown specifically promoted the generation of the long splicing isoform of IKBKG (IKBKG-L). Notably, only IKBKG-L, but not the short variant, stimulated autophagy by promoting the activation of the NF-kB signaling pathway and the transcription of BECN1. These findings suggest that DDX24 regulates autophagy through modulation of IKBKG splicing. Importantly, simultaneous knockdown of IKBKG-L in DDX24-depleted lung cancer cells abrogated the proliferative inhibition induced by DDX24 depletion. This observation indicates that DDX24 controls lung cancer cell proliferation by regulating IKBKG splicing and consequently impacting the autophagic process.

## Results

### DDX24 is elevated in lung cancer and correlated with patients' poor prognosis

**Figure 1:**
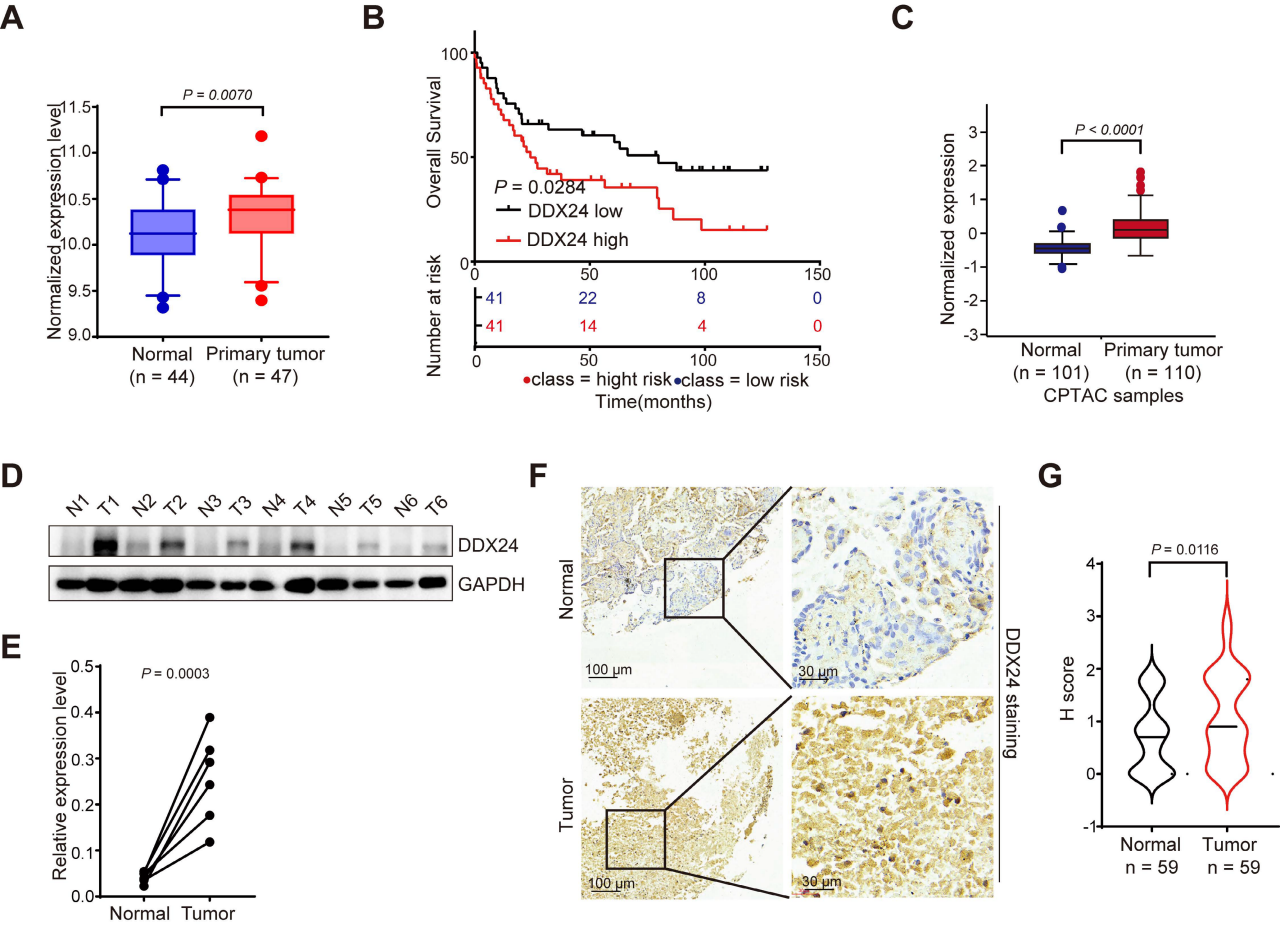
**DDX24 is upregulated in lung cancer and is correlated with patients' poor prognosis. (A)** A boxplot analysis of DDX24 mRNA expression in lung cancer and normal tissues derived from the GSE18842. **(B)** Kaplan-Meier survival analysis for the correlation between DDX24 mRNA level and overall survival in patients with lung cancer from GSE19188 (log-rank test, P = 0.0284). **(C)** DDX24 protein levels in lung cancers from CPTAC dataset. **(D-E)** DDX24 protein levels of six paired lung tumors and adjacent normal tissues from lung cancer patients were analyzed by western blot assay. P value was determined by paired Student t-test. **(F)** Representative images from immunohistochemical staining of DDX24 in lung cancers (n = 59) and normal tissues (n = 59). Scale bars: 100 μm (left) and 30 μm (right). **(G)** IHC staining scores of DDX24 expression were determined.

To investigate the potential role of DDX24 in lung cancer progression, we analyzed its expression levels using multiple datasets. Bioinformatic analysis of the GEO database revealed significantly elevated DDX24 mRNA levels in lung cancer tissues compared to normal lung tissues (Figure 1A). Moreover, DDX24 expression was inversely correlated with overall survival in lung cancer patients (Figure 1B). Consistent with these findings, the Cancer Proteome and Phosphoproteome Atlas (CPPA) database demonstrated increased DDX24 protein abundance in lung cancer tissues (Figure 1C). To further validate these observations, we performed Western blot analysis on clinical lung cancer samples, confirming elevated DDX24 protein levels compared to matched normal controls (Figure 1D-1E). Immunohistochemical staining of tissue microarrays containing lung cancer and adjacent normal tissues (n = 59) independently corroborated these results, demonstrating increased DDX24 immunoreactivity in tumor specimens (Figure 1F-1G). Collectively, our data strongly support the notion that DDX24 is overexpressed in lung cancer and its elevated expression is associated with poor patient prognosis, implicating DDX24 as a potential driver of lung cancer progression.

### Loss of DDX24 suppresses lung cancer progression

**Figure 2:**
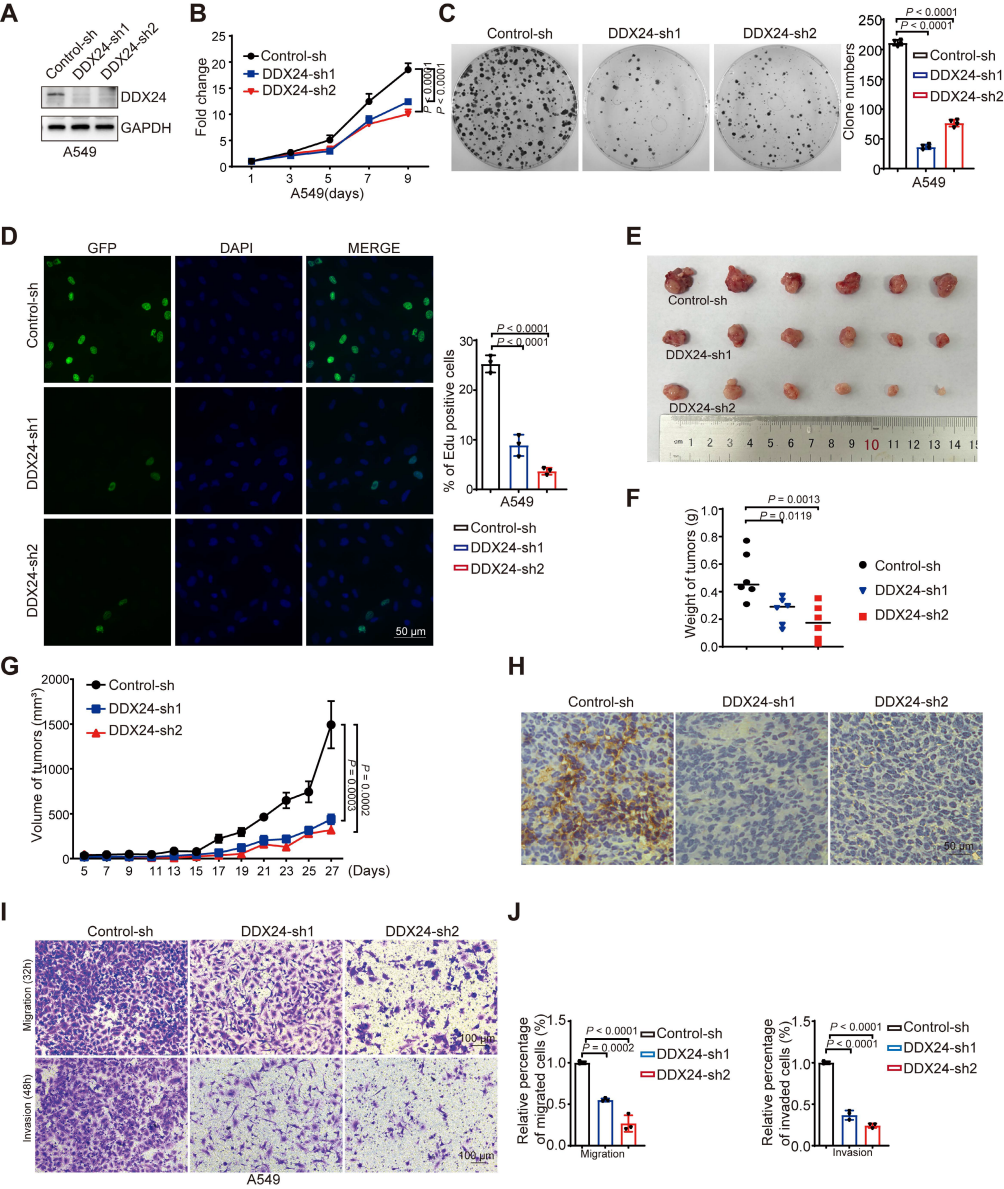
** DDX24 is associated with the progression of lung cancer**. **(A)** Western blot analysis for DDX24 expression in A549 cells infected with two independent shRNAs targeting DDX24 or a control shRNA. **(B)** CCK8 assays were performed to determine cell growth after DDX24 depletion in A549 cells. P values were determined by two-way repeated measures ANOVA. **(C)** Colony formation assays using A549 cells with stable depletion of DDX24. Representative pictures of the whole plates from triplicate experiments are shown. The mean ± SD of colony numbers was plotted, with P values calculated by one-way ANOVA with Dunnett's multiple comparison test. **(D)** The proliferation abilities of A549 cells with DDX24 depletion were determined by EdU staining assay. Quantification of EdU positive cells were plotted, with P values calculated by one-way ANOVA with Dunnett's multiple comparison test. Scale bar: 50 μm. **(E)** Xenograft tumors were generated using nude mice subcutaneously injected with H1299 cells with DDX24 depletion. Pictures of the tumors removed after 27 days were shown. **(F)** Tumors were weighed and plotted. **(G)** The average sizes of xenograft tumors were measured every 2 days (n = 6, error bars indicate ± SD). P values were determined by two-way repeated measures ANOVA. **(H)** Immunohistochemical analysis of DDX24 in xenografts derived from H1299 cells with DDX24 knockdown. Scale bars: 10 μm. **(I-J)** Effect of DDX24 knockdown on migration and invasion of A549 cells evaluated by transwell assays. Scale bars: 100 μm. P values were determined using one-way ANOVA with Dunnett's multiple comparison test (n = 3).

To elucidate the functional significance of DDX24 in lung cancer, we applied growth curve and colony formation assays and revealed a marked reduction in proliferation and colony forming ability in DDX24-depleted lung cancer cells (Figure 2A-2C, Supplementary Figure 1A-1C). Consistently, Edu assay demonstrated impaired cellular growth in DDX24-knockdown cells (Figure 2D, Supplementary Figure 1D). To further evaluate the *in vivo* role of DDX24 in lung cancer, xenograft tumor models were established using DDX24-knockdown and control lung cancer cells in nude mice. Tumor growth was monitored by measuring tumor weight every other day. At endpoint, tumor size and weight were assessed. Importantly, depleted DDX24 resulted in significantly reduced tumor growth rates and smaller tumor volumes compared to control groups (Figure 2E-2G). Immunohistochemical (IHC) staining analysis of xenograft tumor tissues confirmed a marked reduction of DDX24 in tumors derived from DDX24-deficient cells (Figure 2H). Collectively, these data strongly suggest that DDX24 plays a critical role in promoting lung cancer cell growth and proliferation both *in vitro* and *in vivo*.

To determine the role of DDX24 in lung cancer metastasis, transwell assays were conducted, revealing that DDX24 suppression markedly reduced the migratory and invasive capabilities of lung cancer cells (Figure 2I-2J and Supplementary Figure 1E-1F). Together, these data indicate that DDX24 also participates in the regulation of lung cancer metastasis, which is consistent with previous findings 35.

### DDX24 ablation promotes autophagy by promoting autophagosome formation

**Figure 3:**
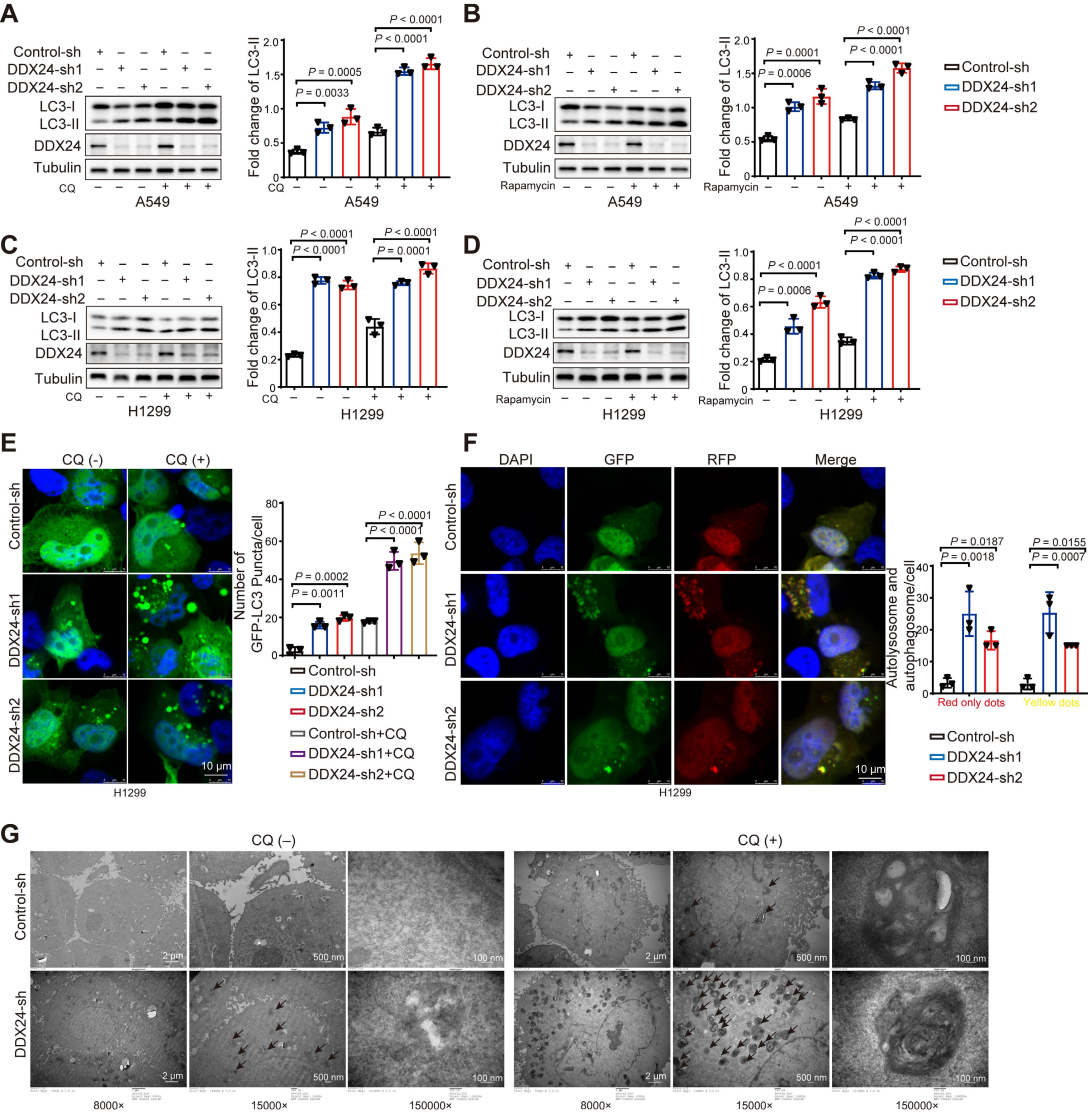
**DDX24 ablation promotes autophagy by promoting autophagosome formation. (A)** The protein levels of LC3 and DDX24 were examined in A549 cells with stable knockdown of DDX24 or control. Cells were treated without or with CQ (40 μM for 2 hrs). **(B)** The protein levels of LC3 and DDX24 were measured in A549 cells with stable knockdown of DDX24 or control. Cells were treated without or with Rapamycin. **(C)** The protein levels of LC3 and DDX24 were determined in H1299 cells with stable knockdown of DDX24 or control. Cells were treated without or with CQ (40 μM for 2 hrs). **(D)** The protein levels of LC3 and DDX24 were examined in H1299 cells with stable knockdown of DDX24 or control. Cells were treated without or with Rapamycin. **(E)** H12999 cells with stable DDX24 depletion or control were transfected with GFP-LC3. 24 hours later, cells were treated with or without CQ for 4 hrs. Three experiments were performed and the number of GFP-LC3 puncta per cell are represented with mean ± SD.** (F)** H12999 cells with stable knockdown of DDX24 were transfected with GFP-mRFP-LC3 to examine the expression of GFP and RFP by confocal microscope. Three experiments were carried out with mean ± SD of the number of autophagosomes (yellow dots) and autolysosomes (red only dots) per cell plotted. The P values were calculated by t-test in all panels puncta per cell are represented with mean ± SD.** (G)** The autophagosomes of A549 cells with stable knockdown of DDX24 were examined with transmission electron microscopy (TEM). Autophagosomes was indicated by black arrows.

Our preliminary data suggested a potential involvement of DDX24 in autophagy regulation. To further investigate this, we generated stable DDX24 knockdown A549 cells. We found a marked increase in LC3-II levels in DDX24 depleted cells as compared to controls (Figure 3A), indicative of elevated autophagosome numbers. To differentiate between enhanced autophagosome formation and impaired autophagic flux, we treated cells with chloroquine (CQ), a lysosomal inhibitor. The persistent increase in LC3-II levels under these conditions suggested that DDX24 primarily promotes autophagosome formation (Figure 3A).

To exclude a role for the canonical autophagy regulator mTORC1, rapamycin treatment was employed. The continued induction of autophagy in DDX24-deficient cells under mTORC1 inhibition indicated that DDX24 knockdown promoted autophagy independently of mTORC1 pathway (Figure 3B). To assess the involvement of p53 in autophagy regulation, we established a DDX24 knockdown H1299 cells. Importantly, autophagy was also elevated in this p53-deficient model, suggesting that such regulation by DDX24 is independently of p53 (Figure 3C-3D).

We further transfected H1299 cells with GFP-LC3 reporter and analyzed by fluorescence microscopy. Consistently, a significant increase in GFP-LC3 puncta was observed in DDX24 depleted cells (Figure 3E), confirming elevated autophagosome numbers. To dynamically monitor autophagosome formation and degradation, we applied the mRFP-GFP-LC3 reporter system. In this system, autophagosomes exhibit yellow fluorescence (from combined red and green signals) while autolysosomes display red-only fluorescence due to GFP quenching in the acidic lysosomal environment. DDX24 knockdown resulted in an increase in both yellow and red fluorescent puncta (Figure 3F), indicating enhanced autophagosome formation and autophagic flux.

To further confirm these findings, electron microscopy analysis demonstrated a significant increase in autophagosome number in DDX24-deficient lung cancer cells (Figure 3G). Moreover, treatment with CQ led to a further accumulation of autophagosomes in DDX24 knockdown cells, providing additional evidence that loss of DDX24 primarily regulates autophagy through the promotion of autophagosome formation.

### DDX24 regulates alternative splicing of autophagy-related genes

**Figure 4:**
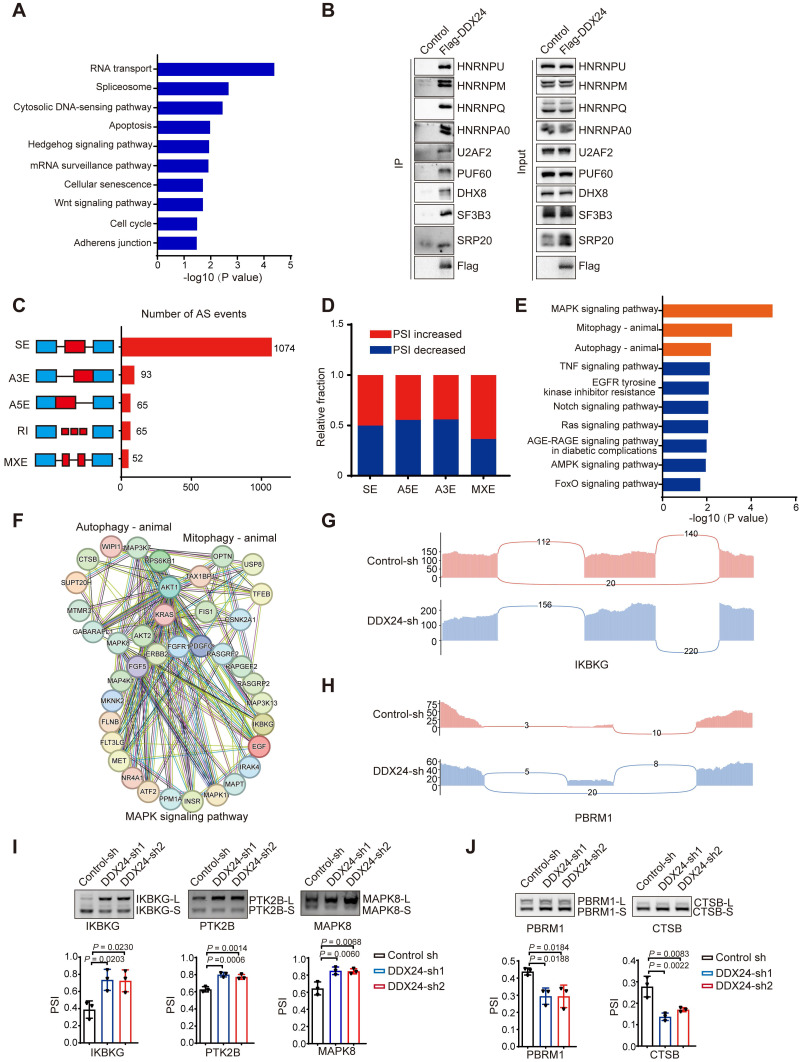
** DDX24 regulates alternative splicing of autophagy-related genes. (A)** Gene ontology analyses of events in the DDX24-immunoprecipitation-mass spectrometry. **(B)** Immunoprecipitation assays was performed for validation of the mass spectrometry data. **(C)** Quantification of the different AS events affected by DDX24. **(D)** The relative fraction of each AS event positively or negatively regulated by DDX24. **(E)** KEGG analysis of DDX24-regulated AS targets. **(F)** Functional association network of DDX24-regulated AS targets were analyzed using the STRING database, and subgroups are marked according to their functions. **(G-H)** Examples of alternative exons affected by DDX24. Genes were chosen to represent both an increase and a decrease of PSI, and the numbers of exon junction reads are indicated. **(I-J)** Selected AS events were validated by RT-PCR. The mean ± SD of relative PSI changes from triplicate experiments were plotted with P values calculated by one-way ANOVA.

To elucidate the molecular mechanisms underlying DDX24-mediated autophagy regulation and its impact on lung cancer progression, we performed RNA-sequencing using DDX24 depleted lung cancer cells. Bioinformatics analysis revealed a critical role for DDX24 in splicing regulation, affecting the splicing of numerous autophagy- and tumor-related genes. DDX24, primarily known as an RNA helicase, has received relatively limited attention for its potential role as a splicing regulatory protein. Our hypothesis that DDX24 functions in this capacity is supported by its involvement in spliceosome regulation, as indicated by Mass spectrometry data following DDX24 immunoprecipitation (Figure 4A). Immunoprecipitation assays further revealed that DDX24 interacts with various splicing-related proteins, including key spliceosome components such as HNRNPU, SF3B3, U2AF2, and PUF60 (Figure 4B). These findings highlight DDX24's role as a splicing regulatory protein, contributing to the regulation of proliferation and autophagy in lung cancer cells. Further analysis indicated that DDX24 predominantly modulated cassette exon splicing (Figure 4C), with no apparent bias in percent spliced in (PSI) value changes (Figure 4D). KEGG pathway enrichment analysis of differentially spliced genes implicated DDX24 in the regulation of multiple signaling pathways, including MAPK signaling pathway, mitophagy, and autophagy (Figure 4E). Protein-protein interaction network analyses of key genes within these pathways further highlighted the complexity of DDX24's regulatory role (Figure 4F). Detailed analysis identified IKBKG and PBRM1 as specific targets of DDX24-mediated splicing regulation (Figure 4G-4H). Additional Gene Set Enrichment Analysis (GSEA) was conducted on splicing events identified in transcriptome sequencing data following DDX24 knockdown. The analysis revealed that DDX24 depletion significantly impacts the alternative splicing of genes involved in metastasis-related pathways, including Epithelial-Mesenchymal Transition (EMT) (Supplementary Figure 2A-2C).

To identify specific splicing events regulated by DDX24, we focused on autophagy-related genes. RT-PCR validation confirmed that DDX24 controls the splicing of multiple genes, including the autophagy-associated gene IKBKG. Our results demonstrated that DDX24 knockdown promoted the generation of longer splicing variants of several genes, including IKBKG, PTK2B, and MAPK8 (Figure 4I), while simultaneously facilitating the production of shorter splicing variants of genes such as PBRM1 and CTSB (Figure 4J). Given the critical role of IKBKG in NF-kB activation and the notable splicing changes we focus on elucidating the functional consequences of these IKBKG splicing variants (full length IKBKG and the short isoform of IKBKG without exon 7) on autophagy and the precise mechanisms by which DDX24 regulates IKBKG splicing.

### DDX24 depletion promotes autophagy by stimulating IKBKG-L-mediated activation of the NF-kB pathway and the elevated transcription of BECN1

**Figure 5:**
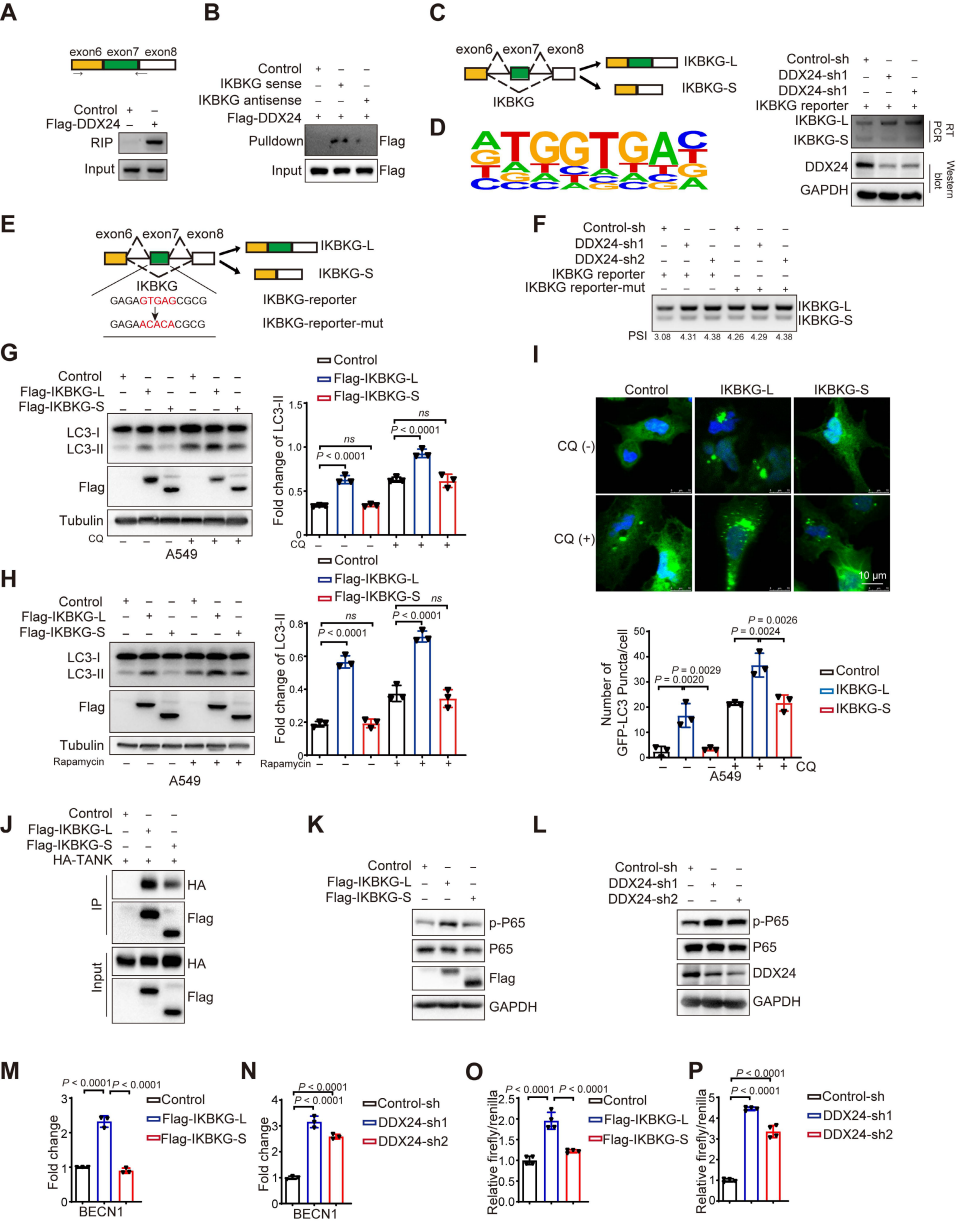
** DDX24 depletion promotes autophagy by stimulating the production of the IKBKG long isoform. (A)** Binding of IKBKG pre-mRNAs with DDX24 was examined by RNA-immunoprecipitation assay in cells exogenously expressed flag-DDX24 or control vector. **(B)** The streptavidin beads-immobilized tRSA-tagged RNA truncations were incubated with cell lysates overexpression Flag-DDX24, and an RNA pull-down assay was performed to check the binding of IKBKG pre-mRNAs with DDX24. **(C)** IKBKG splicing reporter was expressed in cells with stable knockdown of DDX24 or control to examine the splicing change of IKBKG-reporter. The protein levels of DDX24 were also measured. Representative gel and blots were shown. **(D)** Putative DDX24 binding sites. **(E)** The schematic of IKBKG-reporter and IKBKG-reporter-mut. **(F)** IKBKG-reporter or IKBKG-reporter-mut was expressed in cells with stable knockdown of DDX24 or control to examine the splicing change of IKBKG-reporter and IKBKG-reporter-mut. Representative gel and blots were shown. **(G)** The protein levels of LC3 and flag were examined in A549 cells with stable overexpression flag-IKBKG-L, flag-IKBKG-S or control. Cells were treated without or with CQ.** (H)** The protein levels of LC3 and flag were examined in A549 cells with stable overexpression flag-IKBKG-L, flag-IKBKG-S or control. Cells were treated without or with Rapamycin. **(I)** A549 cells with stable overexpression flag-IKBKG-L, flag-IKBKG-S or control were transfected with GFP-LC3. 24 hours later, cells were treated with or without CQ for 4 hrs. Three experiments were performed and the number of GFP-LC3 puncta per cell are represented with mean ± SD. **(J)** Co-immunoprecipitation assays were carried out with anti-Flag antibody and the precipitated complexes were measured by western blotting. **(K-L)** Western blot analysis for P-P65 and P65 expression in cells overexpression Flag-IKBKGL/S (K) or DDX24 knockdown (L). **(M-N)** The mRNA expression of BECN1 was determined in cells overexpression Flag-IKBKGL/S (M) or DDX24 knockdown (N) by qRT-PCR. **(O-P)** BECN1-dual-Luciferase Reporter was transiently transfected into cells overexpression Flag-IKBKGL/S (O) or DDX24 knockdown (P). The relative luciferase activities were determined by calculating the ratio of firefly-luciferase over Renilla-luciferase activities.

To unravel the molecular mechanisms underlying DDX24-mediated splicing of IKBKG, we used the primers described in the methods section and shown as the upper part in Figure 5A to perform RNA immunoprecipitation (RIP) assays, demonstrating a direct interaction between DDX24 and IKBKG mRNA (Figure 5A). Additionally, RNA pulldown experiments further corroborated that DDX24 binds directly to IKBKG mRNA, validating the results of the RIP assay (Figure 5B). To further characterize the regulatory role of DDX24 in IKBKG splicing, we constructed an IKBKG splicing reporter that contains key splicing elements of IKBKG pre-mRNA. The reporter design was adapted from previously published studies by our laboratory 23. Briefly, this reporter includes the alternatively spliced exon 7, its adjacent exon 6 and exon 8, and the intronic regions between these exons. By incorporating both splice sites and branch points, this design faithfully replicates the splicing events (Figure 5C, left). As expected, DDX24 depletion significantly promoted the production of IKBKG-L, the canonical long IKBKG isoform (Figure 5C, right). Homer motif analysis was applied to DDX24 enhanced cross-linking and immunoprecipitation (eCLIP)-seq data (ENCODE accession ID ENCFF142OCF). This analysis identified the potential binding motif of DDX24, allowing us to locate its specific binding site near the exon7 within the IKBKG gene (Figure 5D). To pinpoint the DDX24 binding sites, a mutant reporter with disrupted DDX24 binding motifs was generated (Figure 5E). Interestingly, mutations of the DDX24 binding sites abolished the DDX24 depletion-induced elevated levels of IKBKG-L (Figure 5F, lane 4-6), suggesting that DDX24 specifically binds to exon 7 of IKBKG to regulate its splicing.

To further investigate whether other splicing isoforms of IKBKG are also regulated by DDX24, we analyzed the splicing isoforms that produced by alternative splicing in the CDS region using the UCSC Genome Browser. Isoform-specific primers were designed, and RT-PCR analysis revealed that DDX24 does not affect the overall expression level of IKBKG mRNA (Supplementary Figure 3A). In addition, the production of another two splicing isoforms (NM_001377315.1 and NM_001145255.4) was not influenced by DDX24 either (Supplementary Figure 3A). These findings confirmed that DDX24 depletion promotes the production of the canonical long isoform of IKBKG (NM_001321397.3) while suppresses the generation of the short isoform without exon 7 (NM_001377314.1).

To determine the function differences between IKBKG splicing variants in autophagy regulation, A549 cells overexpressing either the long or short IKBKG isoform were generated. Importantly, overexpression of the long IKBKG isoform significantly increased LC3-II levels compared to controls, indicative of enhanced autophagy, whereas the short isoform had no effect on autophagy (Figure 5G and Supplementary Figure 3B). To further explore the mechanism, cells were treated with CQ and rapamycin to inhibit lysosomal function and mTORC1 signaling, respectively. Under these conditions, the long IKBKG isoform continued to promote LC3-II accumulation, while the short isoform remained without effect (Figure 5G-5H and Supplementary Figure 3B-3C). To visualize autophagosome formation, GFP-LC3-transfected cells were analyzed by fluorescence microscopy. Overexpression of the long IKBKG isoform resulted in elevated autophagosome formation, however, the short isoform had no impact (Figure 5I and Supplementary Figure 3D). Altogether, these data demonstrate that the long IKBKG isoform, but not the short isoform, promotes autophagy through stimulating autophagosome formation, suggesting that DDX24-mediated upregulation of the long IKBKG isoform likely contributes to the observed increase in autophagy.

To study why the long isoform of IKBKG promotes autophagy while the short isoform does not, we performed a detailed analysis of the amino acid sequence within exon 7 of IKBKG, revealing that the short isoform of IKBKG lacks a domain critical for interaction with TANK 33. Co-immunoprecipitation assays demonstrated that the long splicing isoform of IKBKG interacts significantly more strongly with TANK than the short splicing isoform (Figure 5J). Since the interaction between IKBKG and TANK is essential for the activating the NF-kB signaling pathway 33,34, we further examined the effects of the two isoforms on this pathway. The long isoform of IKBKG was found to significantly promotes p65 phosphorylation, a function substantially diminished in the short isoform (Figure 5K). Additionally, we examined the impact of DDX24 knockdown on the NF-kB signaling pathway. Notably, DDX24 knockdown also increases the phosphorylation of p65 (Figure 5L).

Although the NF-kB signaling pathway is known to play a key role in autophagy 36-38, we sought to identify specific effector genes involved in autophagy that are activated by the long isoform of IKBKG. Using qRT-PCR to screen autophagy-related genes, we observed that the overexpression of the long isoform of IKBKG significantly increased the expression of BECN1, whereas the short isoform of IKBKG failed to induce BECN1 expression (Figure 5M). Similarly, knockdown of DDX24 also resulted in the upregulation of BECN1 expression (Figure 5N). Further validation using luciferase reporter assays confirmed that both the overexpression of the long isoform of IKBKG and the knockdown of DDX24 activate BECN1 transcription (Figure 5O-5P). These findings suggest that DDX24 depletion promotes autophagy by stimulating the production of the long isoform of IKBKG, which activates the NF-kB signaling pathway and drives BECN1 transcription.

### Loss of DDX24 inhibits lung cancer progression by stimulating IKBKG-L-mediated autophagy

**Figure 6:**
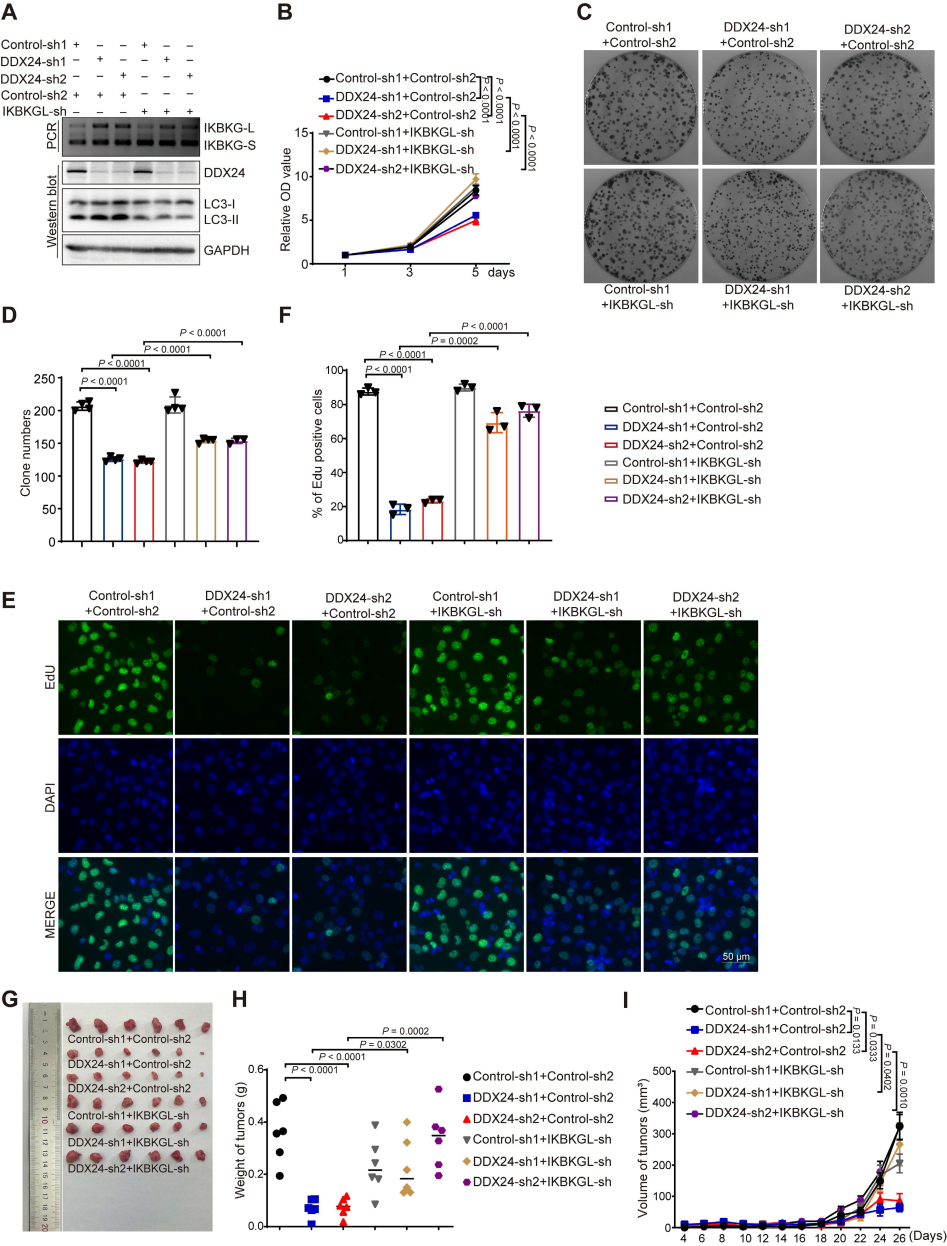
** Loss of DDX24 inhibits lung cancer progression by stimulating IKBKG-L-mediated autophagy. (A)** The mRNA levels of IKBKG-L or IKBKG-S were examined by RT-PCR in A549 cells with stable DDX24 knockdown in the presence or absence of IKBKG-L. The protein levels of LC3 and DDX24 were examined by western-blot assays in A549 cells with stably depleted DDX24 with or without IKBKG-L. **(B)** CCK8 assays were performed to determine cell growth in A549 cells with stable DDX24 depletion with or without IKBKGL. P values were determined by two-way repeated measures ANOVA. **(C-D)** Colony formation assays using A549 cells with stable knockdown of DDX24 in the presence of absence of IKBKG-L. Representative pictures of the whole plates from triplicate experiments are shown. The mean ± SD of colony numbers was plotted, with P values calculated by one-way ANOVA with Dunnett's multiple comparison test, t-test. **(E-F)** The proliferation abilities of A549 cells with stable depletion of DDX24 in the presence of absence of IKBKG-L. Quantification of EdU positive cells were plotted, with P values calculated by one-way ANOVA with Dunnett's multiple comparison test, t-test. Scale bar: 50 μm.** (G)** Xenograft tumors were generated using nude mice subcutaneously injected with DDX24 knockdown cells in the presence or absence of IKBKG-L. Pictures of the tumors removed after 26 days were shown. **(H)** Tumors were weighed and plotted.** (I)** The average sizes of xenograft tumors were measured every 2 days (n = 6, error bars indicate ± SD). P values were determined by two-way repeated measures ANOVA.

To investigate whether IKBKG-L-mediated autophagy is responsible for depleted DDX24-induced suppression of lung cancer progression, we generated lung cancer cell lines with concurrent knockdown of DDX24 and the long IKBKG isoform. We found that the induction of autophagy by DDX24 depletion was reversed upon knockdown of IKBKG-L, as judged by the reduced level of LC3-II (Figure 6A and Supplementary Figure 4A). Functional assays revealed that the growth inhibitory effects of DDX24 depletion were rescued by simultaneous knockdown of IKBKG-L, as evidenced by reduced proliferation, colony formation, and EdU incorporation (Figure 6B-6F, Supplementary Figure 4B-4F). To further explore the tumor-suppressive effects of DDX24 depletion *in vivo*, driven by the increased production of the long isoform of IKBKG, we conducted xenograft mouse experiments using cell lines with simultaneous silencing of DDX24 and IKBKG-L. The results demonstrated that the reduction in tumor growth induced by DDX24 knockdown was significantly attenuated when IKBKG-L was concurrently silenced *in vivo* (Figure 6G-6I). Collectively, these findings suggest that loss of DDX24 suppresses lung cancer progression by stimulating autophagy through promoting the production of IKBKG-L.

### The long IKBKG isoform is positively associated with improved survival in lung cancer patients

**Figure 7:**
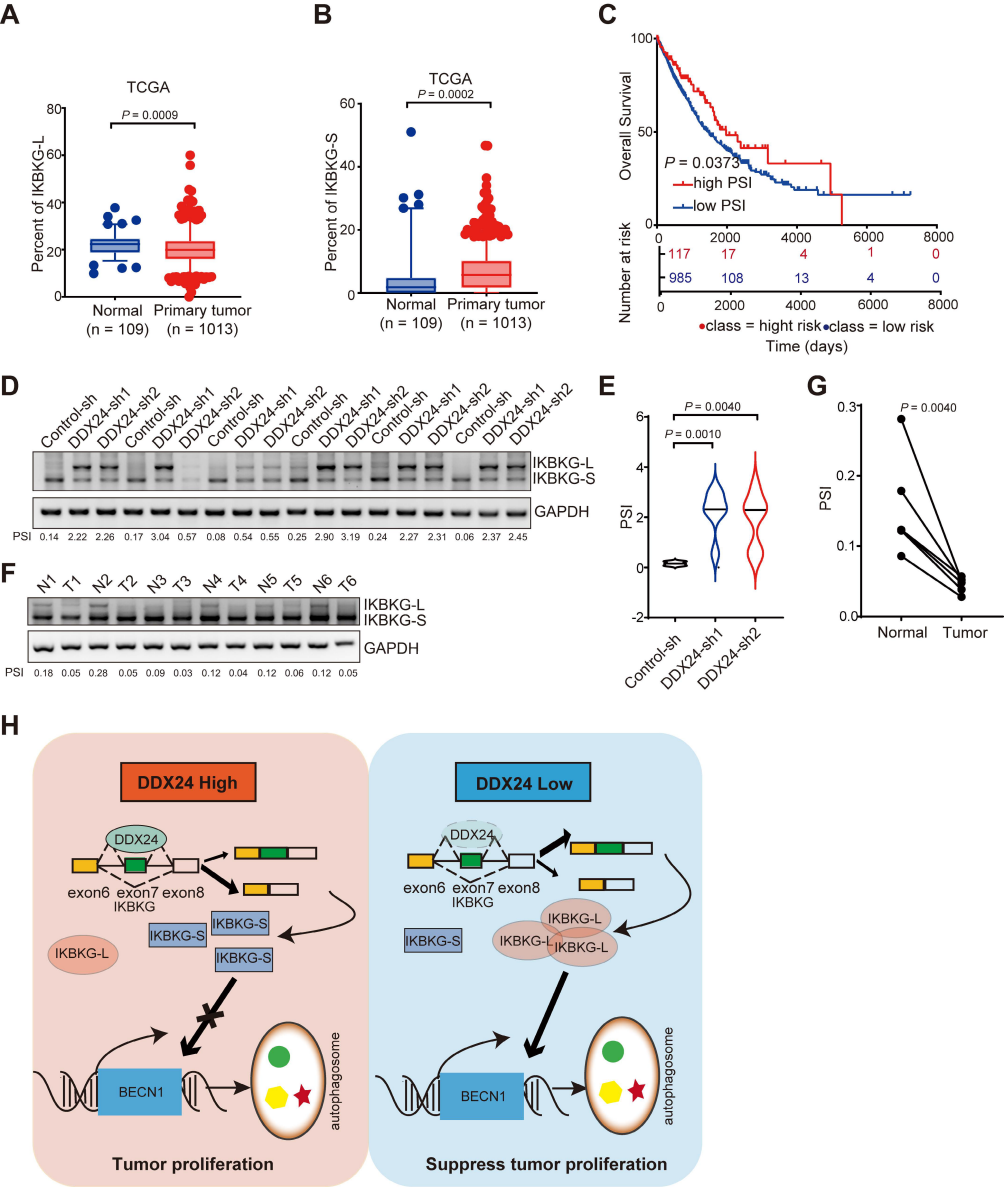
** Higher PSI of IKBKG closely associated with improved survival in lung cancer patients. (A)** The percent of IKBKG-L was analyzed in lung cancer based on TCGA database. **(B)** The percent of IKBKG-S was analyzed in lung cancer based on TCGA database.** (C)** Kaplan-Meier survival analysis for the correlation between the splicing alteration of IKBKG and overall survival in patients with lung cancer from TCGA database (log-rank test, P = 0.0373). **(D-E)** IKBKG PSI in tumor tissues obtained from xenograft experiments in nude mice. P value was determined by paired Student t-test. **(F-G)** PSI of IKBKG of six paired lung tumors and adjacent normal tissues from lung cancer patients were analyzed by RT-PCR. P value was determined by paired Student t-test. **(H)** The schematic of loss of DDX24 inhibits lung cancer progression by stimulating IKBKG splicing-mediated autophagy.

To comprehensively assess the clinical relevance of IKBKG splicing in lung cancer, we conducted an analysis of the TCGA dataset. Our findings indicate a decreased proportion of IKBKG-L and an increased proportion of IKBKG-S in lung cancer specimens compared to normal tissue (Figures 7A and 7B). Furthermore, a robust correlation was observed between a reduction in IKBKG Percent Spliced In (PSI) and unfavorable prognosis in lung cancer patients (Figure 7C).

To further elucidate the impact of DDX24 on IKBKG splicing, we examined the splicing of IKBKG in tumor tissues from xenograft models. A significant increase of IKBKG-L was observed in tumors with DDX24 depletion (Figure 7D-7E). To validate these findings in a clinical setting, we analyzed IKBKG splicing in paired lung cancer and adjacent normal tissue samples. RT-PCR assay revealed a marked decrease of IKBKG-L (reduced PSI) in tumor tissues compared to normal tissues (Figure 7F-7G), suggesting a potential role for IKBKG alternative splicing in lung cancer progression.

Collectively, our results demonstrate that reduced DDX24 expression correlates with increased IKBKG-L production, which subsequently promotes autophagy and suppresses lung cancer cell proliferation (Figure 7H).

## Discussion

Lung cancer remains a leading cause of cancer-related mortality, necessitating the identification of novel therapeutic targets. Our research has focused on the role of RNA-binding proteins (RBPs) in tumorigenesis and tumor progression. A comprehensive analysis of RBPs implicated DDX24 in autophagy regulation, prompting a deeper investigation into its role in lung cancer. Consistent with its potential oncogenic function, DDX24 protein levels were significantly elevated in lung cancer tissues compared to normal tissues. Functional studies using DDX24-deficient lung cancer cell lines demonstrated impaired tumor growth both *in vitro* and *in vivo*. These findings establish DDX24 as a promising therapeutic target for lung cancer.

As a member of the DEAD box RNA helicase family, DDX24 plays a critical role in RNA metabolism. Its involvement in cancer is multifaceted, previous studies have linked DDX24 to liver cancer progression through stabilization of LAMB1 mRNA 39-40, and gastric cancer growth 41. In addition, DDX24 modulates p53 activity 26, maintains kinase activity 42. These findings highlight the diverse functions of DDX24 and its potential as a key player in multiple cancer types.

Autophagy, a critical cellular catabolic process, is implicated in diverse cellular functions including protein and organelle turnover. Its role in tumorigenesis is complex and context-dependent. Our previous work demonstrated that the RNA binding protein SRSF1 influences lung cancer progression through regulating Bcl-x splicing and autophagy 43. Among a panel of RBPs, DDX24 depletion was associated with enhanced autophagic activity. To elucidate the underlying mechanism, we generated DDX24-deficient lung cancer cell lines. Western blot analysis revealed increased LC3-II levels, a hallmark of autophagy, following DDX24 depletion. To distinguish between increased autophagosome formation and impaired autophagic flux, we employed lysosomal inhibition and a dual-fluorescence tf-LC3 reporter system. Our results indicate that DDX24 primarily regulates autophagy by promoting autophagosome biogenesis.

Our findings demonstrate that DDX24 suppression promotes autophagy and inhibits lung cancer progression. To identify downstream effectors, transcriptome sequencing analysis revealed DDX24's role in regulating alternative splicing of autophagy-related genes. Notably, DDX24 knockdown increased exon 7 inclusion in the IKBKG transcript. As a core component of the IKK complex, IKBKG is pivotal in NF-kB activation and has been implicated in both cancer and autophagy. The exon 7-encoded region is predicted to influence IKBKG's interaction with TANK, potentially impacting its function in NF-kB signaling and autophagy. To further investigate the interaction between IKBKG splicing isoforms and TANK, we conducted co-immunoprecipitation assays. The results demonstrated that the canonical long splicing isoform of IKBKG exhibited a substantially stronger interaction with TANK compared to the short isoform. Additionally, overexpression of the long isoform significantly enhanced p65 phosphorylation and proved more effective than the short isoform in activating the NF-kB signaling pathway.

The nuclear factor-kB (NF-kB)/Rel proteins include NF-kB2 p52/p100, NF-kB1 p50/p105, c-Rel, RelA/p65, and RelB. These multifunctional dimeric transcription factors are critical regulators of gene expression, influencing a variety of biological processes. The IKK complex, comprising IKKβ, IKKα, and IKBKG, plays a central role in activating the NF-kB/Rel complex. Upon activation, NF-kB/Rel complexes undergo post-translational modifications—such as phosphorylation, acetylation, and glycosylation—that are essential for their nuclear translocation. Within the nucleus, they function independently or synergistically with other transcription factors, including AP-1, Ets, and Stat, to drive the expression of target genes 44-46. Our findings indicate that the knockdown of DDX24 triggers the NF-kB signaling pathway by promoting the production of the long splicing isoform of IKBKG, resulting in the transcription of BECN1. This demonstrate that DDX24 is involved in autophagy by modulating the expression of BECN1, which is consistent with our previous findings that the effect of DDX24 on autophagy is independent of the mTORC1 and p53 signaling pathways.

Previous studies have suggested that DDX24 may play a role in the metastatic process of lung cancer 35. Building on this, we performed transwell assay, and found that DDX24 depletion does inhibit migration and invasiveness of lung cancer cells. Further Gene Set Enrichment Analysis (GSEA) of sequencing data following DDX24 knockdown revealed that DDX24 likely regulates the alternative splicing of genes involved in several metastasis-related pathways, including epithelial-mesenchymal transition (EMT). The precise mechanisms by which DDX24 influences lung cancer metastasis through alternative splicing will be addressed in our future studies.

Our results suggest that DDX24 modulates lung cancer cell proliferation by regulating IKBKG alternative splicing and subsequent autophagy induction. While these findings provide novel insights into the complex interplay between autophagy, splicing, and cancer, further studies are required to elucidate the precise mechanisms underlying the functional differences between IKBKG splice variants in the context of lung cancer.

## Methods

### Cell culture and treatment

Lung cancer cell lines derived from humans (designated H1299 and A549) were procured from the American Type Culture Collection. These cells were cultured in mediums specific to their needs (RPMI 1640 for H1299 and F12K for A549) each enriched with 10% Fetal Bovine Serum (FBS). To establish a stable reduction of DDX24 expression in H1299 and A549 cells, we employed lentiviral vectors. This process entailed transfecting 293T cells using pLKO.1-DDX24 plasmid (with pLKO.1 empty vector serving as a control), along with PAX2 and PMD2, adhering strictly to the supplier's guidelines. Subsequently, the viral-laden supernatant was harvested post-centrifugation to eliminate cellular impurities. Thereafter, H1299 and A549 cells were exposed to these viral particles, and those cells successfully incorporating the lentivirus were selectively grown in the presence of 2 μg/ml puromycin over a period of five days. The surviving cells, now stably expressing the knockdown, were continually propagated in media supplemented with 1 μg/ml puromycin within a 37 °C, 5% CO_2_ humidified incubator. Prior to proceeding with further analyses, the stable knockdown in all generated cell lines was validated through Western blot assays.

### Western blot

Cells were collected using RIPA lysis buffer, which was mixed with 1 mM sodium orthovanadate, 1 mM protease inhibitor cocktail, and 1 mM PMSF to preserve protein integrity. Cellular debris was eliminated through a centrifugation step. Thereafter, equivalent quantities of the resulting lysates were subjected to SDS-polyacrylamide gel electrophoresis (SDS-PAGE), followed by electrotransfer onto a nitrocellulose membrane to prepare for immunoblotting. For the detection of specific proteins, the following primary antibodies were employed: DDX24 antibody from Proteintech (catalog number 15769-1-AP), LC3B antibody sourced from Sigma (catalog number L7543), Flag antibody by Sigma (catalog number F1802), GAPDH antibody provided by Abways (catalog number AB0037), and Tubulin antibody also from Proteintech (catalog number 11224-1-AP). The binding of these antibodies to their respective targets was visualized utilizing an enhanced chemiluminescence reagent (supplied by Tanon) and captured using a MiniChemi Chemiluminescence Imager system, a product of SageCreation based in Beijing.

### Real-time PCR

RNA was isolated from the cells employing the Trizol reagent, adhering meticulously to the protocol outlined by Invitrogen. Residual genomic DNA present in the total RNA extract (1 microgram) was thoroughly eliminated and the purified RNA was subsequently reverse transcribed into cDNA utilizing the Vazyme RT reagent kit (catalog number RT101-01), with 1 microliter of the synthesized cDNA being utilized for subsequent polymerase chain reaction (PCR) amplification. Quantitative real-time PCR (qPCR) was conducted utilizing the MonAmp ChemoHS qPCR Mix (product code MQ00401S), carried out on the QuantStudio3 Real-Time PCR system. This approach facilitated precise measurement of target gene expression levels in the samples.

### Xenograft assays

The animal experimental procedures were authorized by the Institutional Animal Care and Use Committee at Dalian Medical University, ensuring ethical compliance. In this study, eighteen 4-week-old BALB/c nude mice were acquired from Changsheng Bio-Technology and, in a blinded and randomized manner, allocated into three distinct groups, each consisting of six mice. These mice were then subcutaneously inoculated in their abdomen side with either H1299 cells having undergone DDX24 knockdown or control cells, at a dosage of 3×10^6^ cells per mouse. Over the course of 27 days, the mice were observed visually and the dimensions of the tumors were measured every other day to track their growth. The tumor volume was estimated using the formula 1/2r1^2^r2, (r1 < r2). Upon completion of the 27-day observation period, all mice were humanely euthanized, and their tumors were excised for additional investigations. To ensure accuracy, every data point represented the average of five separate measurements, and these averages were employed for subsequent calculations and analyses.

### Transmission electron microscopy

To conduct an in-depth examination of autophagosome structures, transmission electron microscopy (TEM) was employed. H1299 cells underwent fixation in a solution composed of 2.5% glutaraldehyde dissolved in 0.1 M phosphate-buffered saline (PBS) adjusted to a pH of 7.4, maintained at 4 °C for a duration of 2.5 hours. Post-fixation, the cells were rinsed thrice with 0.1 M PBS and subjected to secondary fixation with 1% osmium tetroxide for an additional 2 hours under refrigeration. Subsequently, the samples underwent a graded dehydration process using a series of ethanol solutions, leading up to infiltration and embedding in Spurr's resin. Ultrathin sections were then sliced from the resin-embedded samples and stained with either uranyl acetate or lead citrate to enhance contrast. These prepared sections were finally inspected and analyzed under the high-resolution capabilities of a JEOL 1200EX transmission electron microscope.

### RIP assay

RNA immunoprecipitation was performed following the previously established protocol. In summary, Flag-DDX24 or control H1299 cells were harvested and fixed with 1% formaldehyde for a period of 10 minutes. Subsequently, the fixation was quenched by treating the samples with a glycine solution for 5 minutes, followed by twice washes with precooled PBS. The harvested cells were lysed with 1 mL of IP lysis buffer (comprising 50 mM Tris-HCl pH 7.5, 0.4 M NaCl, 1 mM EDTA, 1 mM DTT, 0.5% Triton X-100, 10% Glycerol, supplemented with protease inhibitors and RNase inhibitors), and subjected to three cycles of sonication. To eliminate non-specific binding, the lysates were pre-cleared by mixing with Protein A/G agarose beads and non-specific tRNA before proceeding with immunoprecipitation using Anti-Flag M2 Affinity beads. Following six times washes with 1 mL of IP lysis buffer, the beads were suspended in 100 μL of RIP buffer (50 mM Tris-HCl pH 7.5, 0.1 M NaCl, 5 mM EDTA, 10 mM DTT, 0.5% Triton X-100, 10% Glycerol, 1% SDS) and incubated at 70 °C for 45 minutes to reverse the cross-links. Finally, the RNA was isolated using Trizol reagent, converted into cDNA via reverse transcription, and detected through PCR analysis. Primers used as follows:

IKBKG-RIP-F: CTGTGAAAGCCCAGGTGACG

IKBKG-RIP-R: CAGCTTCCTCTTCTCCTCCG

### RNA pulldown

The RNA pull-down template, equipped with a tRNA scaffolded Streptavidin Aptamer (tRSA), was generated by cloning into a pCDNA3-tRSA vector and applied to an *in vitro* transcription 47. For the *in vitro* transcription, the MEGAscript™ T7 kit (Invitrogen AM1333) was employed following the manufacturer's instructions. Following transcription, RNA was extracted according to the previously described method. Streptavidin beads (ThermoFisher Scientific; Catalog No. 11205D) were prepped by washing them twice with 1 mL of lysis buffer (10 mM HEPES, pH 7.0, 200 mM NaCl, 10 mM MgCl2, 1 mM DTT, 1% Triton X-100). The beads were then blocked with 1 mL of lysis buffer supplemented with 5% BSA and 100 µg of tRNA at 4 °C for 1 hour. Cells overexpressing Flag-DDX24 were lysed in lysis buffer (with 1 mM PMSF and 1× protease inhibitor cocktail) and subjected to eight cycles of sonication. The *in vitro* transcribed RNA was denatured at 65 °C for 5 minutes and gradually cooled to room temperature in a solution containing 5× RNA structure buffer (50 mM HEPES, pH 7.0, 50 mM MgCl2). The precleared cell lysate was treated with 5 µL of RNasin (40 U/µL) and combined with the denatured RNAs on a rotating shaker at 4 °C for 1 hour. This mixture was then added to the blocked Streptavidin beads and incubated for 6 hours at 4 °C. Afterward, the beads were washed six times with 1 mL of wash buffer (10 mM HEPES, pH 7.0, 400 mM NaCl, 10 mM MgCl2, 1 mM DTT, 1% Triton X-100, 4 U/mL RNasin, 1 mM PMSF, and 1× protease inhibitor cocktail). The proteins bound to the RNA were finally eluted and subjected to western blot analysis.

### Immunoprecipitation

For the immunoprecipitation procedure, H1299 cells expressing the control vector or Flag-IKBKG-L/S, were transfected with HA-TANK expression constructs using the LipoPlus reagent (Sagecreation). Following a 24-hour incubation period, the cells were harvested and lysed in a buffer solution containing 50 mM Tris/HCl (pH 7.5), 150 mM NaCl, 1 mM EDTA, 1% Triton X-100, 1 mM protease inhibitor cocktail, and 1 mM PMSF. The resulting cell lysates were then incubated with Anti-Flag M2 beads (Sigma) overnight at 4 °C. Subsequently, the beads were washed five times with TBS (50 mM Tris/HCl, pH 7.5, 150 mM NaCl) supplemented with 0.1% Triton X-100, after which the immunoprecipitated complexes were analyzed by immunoblotting using appropriate antibodies.

### Dual-Luciferase reporter assay

To construct the BECN1-dual-Luciferase reporter, the promoter region of the human BECN1 gene was amplified via PCR and subsequently inserted into the pGL3 basic vector. H1299 cells, which had undergone DDX24 knockdown or overexpression of IKBKG-L/S, along with an empty vector control, were co-transfected with the BECN1-dual-Luciferase reporter construct and a Renilla luciferase control plasmid for 24 hours. Luciferase activity was then assessed using a dual-luciferase reporter assay system (Promega, E1910) on a SpectraMax iD3 Multi-Mode Microplate Reader. Firefly luciferase (FLuc) activity was normalized to Renilla luciferase (RLuc) activity to determine the efficiency of reporter gene transcription. The primers utilized are listed below:

BECN1-Nhe1-promoter-F: CTAGCTAGCAGTAGCCAGCTCTTCATTG

BECN1-Xho1-promoter-R: CCGCTCGAGACCATCGCTCTGTCTTCA

### Statistical analysis

All data are presented as the means ± SD from at least three independent experiments. Statistical significance for each experiment was determined by two-tailed unpaired or paired t test, and one-way ANOVA with Dunnett multiple comparisons, two-way repeated measures ANOVA, as appropriate. The groups compared are annotated in figures or Figure legends. Statistical analyses were performed using Prism 8 (Graph Pad).

## Supplementary Material

Supplementary figures.
