# Supplementary material for: Loss of DDX24 inhibits lung cancer progression by stimulating IKBKG splicing-mediated autophagy
Source: Theranostics. 2025 Jan 2;15(5):1879–95. doi: 10.7150/thno.102425 (PMC11780526; doi:10.7150/thno.102425)
Supplement: Supplementary file 1 — Supplementary figures. [file thnov15p1879s1.pdf]

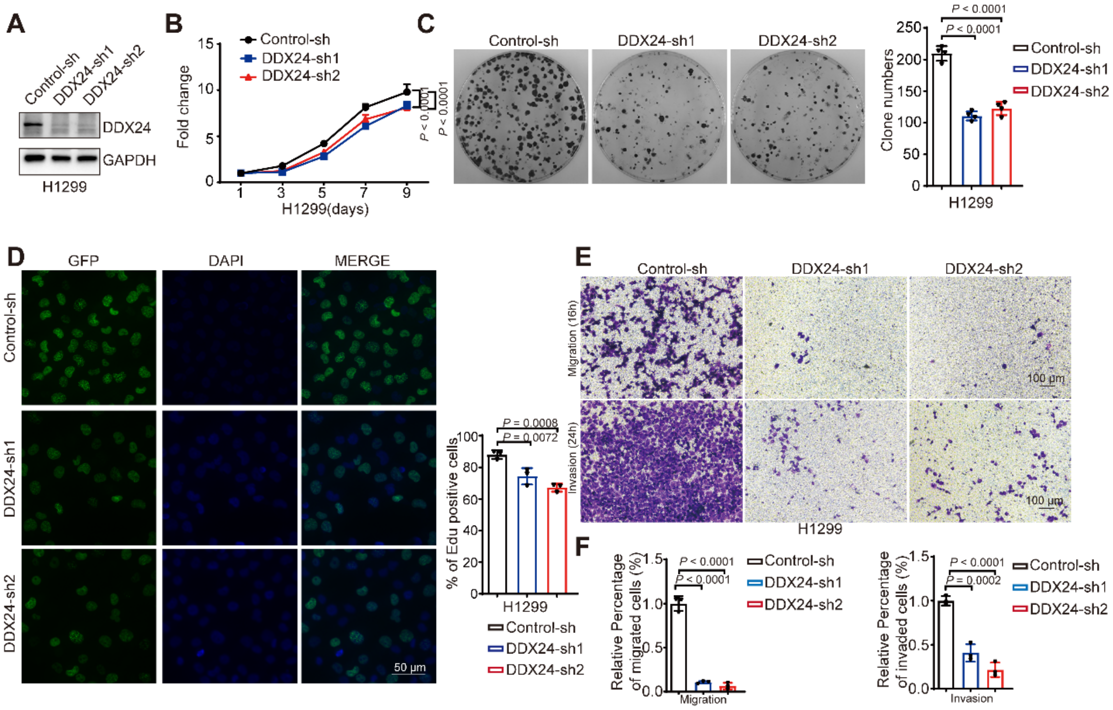

Supplementary Figure 1. DDX24 is associated with the progression of lung cancer. **(A)** Western blot analysis for DDX24 expression in H1299 cells infected with two independent shRNAs targeting DDX24 or a control shRNA. **(B)** CCK8 assays were performed to determine cell growth after DDX24 was knocked down in H1299 cells. P values were determined by two-way repeated measures ANOVA. **(C)** Colony formation assays using H1299 cells with stable depletion of DDX24. Representative pictures of the whole plates from triplicate experiments are shown. The mean ± SD of colony numbers was plotted, with P values calculated by one-way ANOVA with Dunnett's multiple comparison test. **(D)** The proliferation abilities of H1299 cells with DDX24 depletion were determined by EdU staining assay. Quantification of EdU positive cells were plotted, with P values calculated by one-way ANOVA with Dunnett's multiple comparison test. Scale bar: 50 μm. **(E-F)** Effect of DDX24 knockdown on migration and invasion of H1299 cells evaluated by transwell assays. Scale bars: 100 μm. P values were determined using one-way ANOVA with Dunnett's multiple comparison test (n = 3).

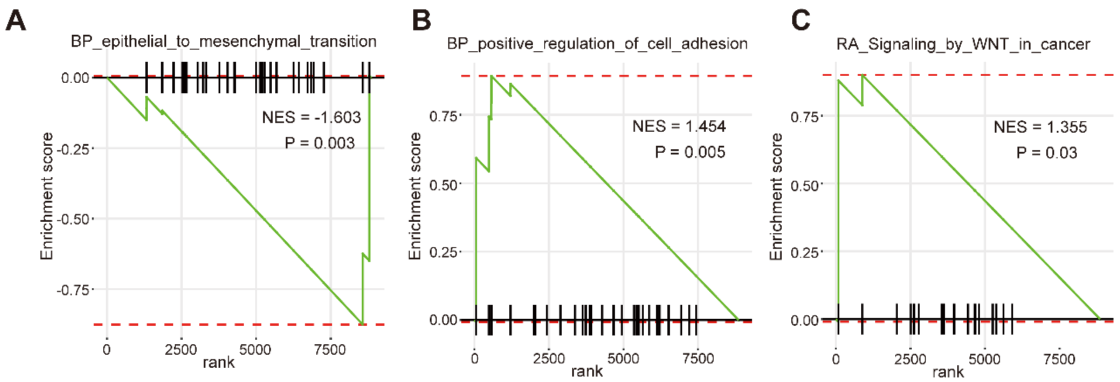

Supplementary Figure 2. DDX24 regulates metastasis-associated splicing events in lung cancer cells.

(A-C) GSEA showing significant enrichment of EMT (G), cell adhesin (H) and WNT (I) pathway in DDX24 knockdown cells compared to those with control sh.

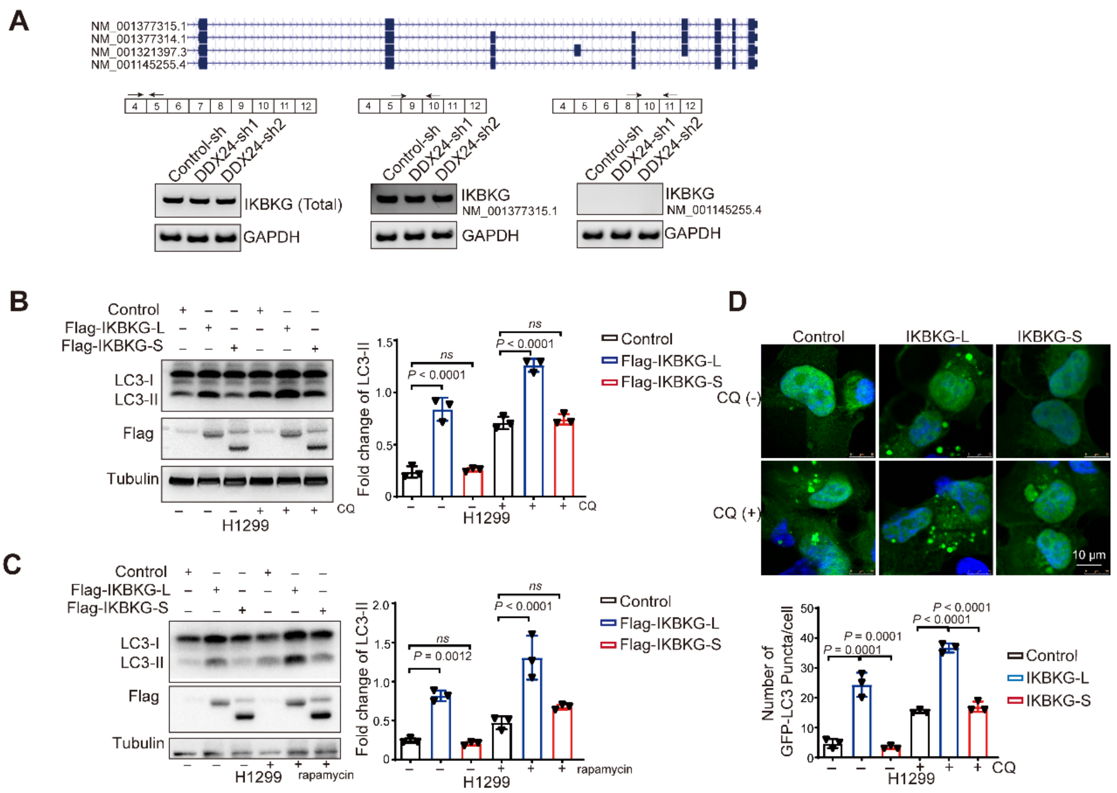

Supplementary Figure 3. DDX24 knockdown promoted autophagy by stimulating the production of the IKBKG long splicing isoform. (A) The expression levels of total IKBKG mRNA and its various splice variants in DDX24 knockdown cells were detected using RT-PCR. **(B)** The protein levels of LC3 and flag were examined in H1299 cells with stable overexpression flag-IKBKG-L, flag-IKBKG-S or control. Cells were treated without or with CQ (40 μM for 2 h). **(C)** The protein levels of LC3 and flag were examined in H1299 cells with stable overexpression flag-IKBKG-L, flag-IKBKG-S or control. Cells were treated without or with Rapamycin. **(D)** H1299 cells with stable overexpression flag-IKBKG-L, flag-IKBKG-S or control were transfected with GFP-LC3. Twenty-four hours later, cells were treated with or without CQ for 4 h. Three experiments were performed and the number of GFPLC3 puncta per cell are represented with mean ± SD.

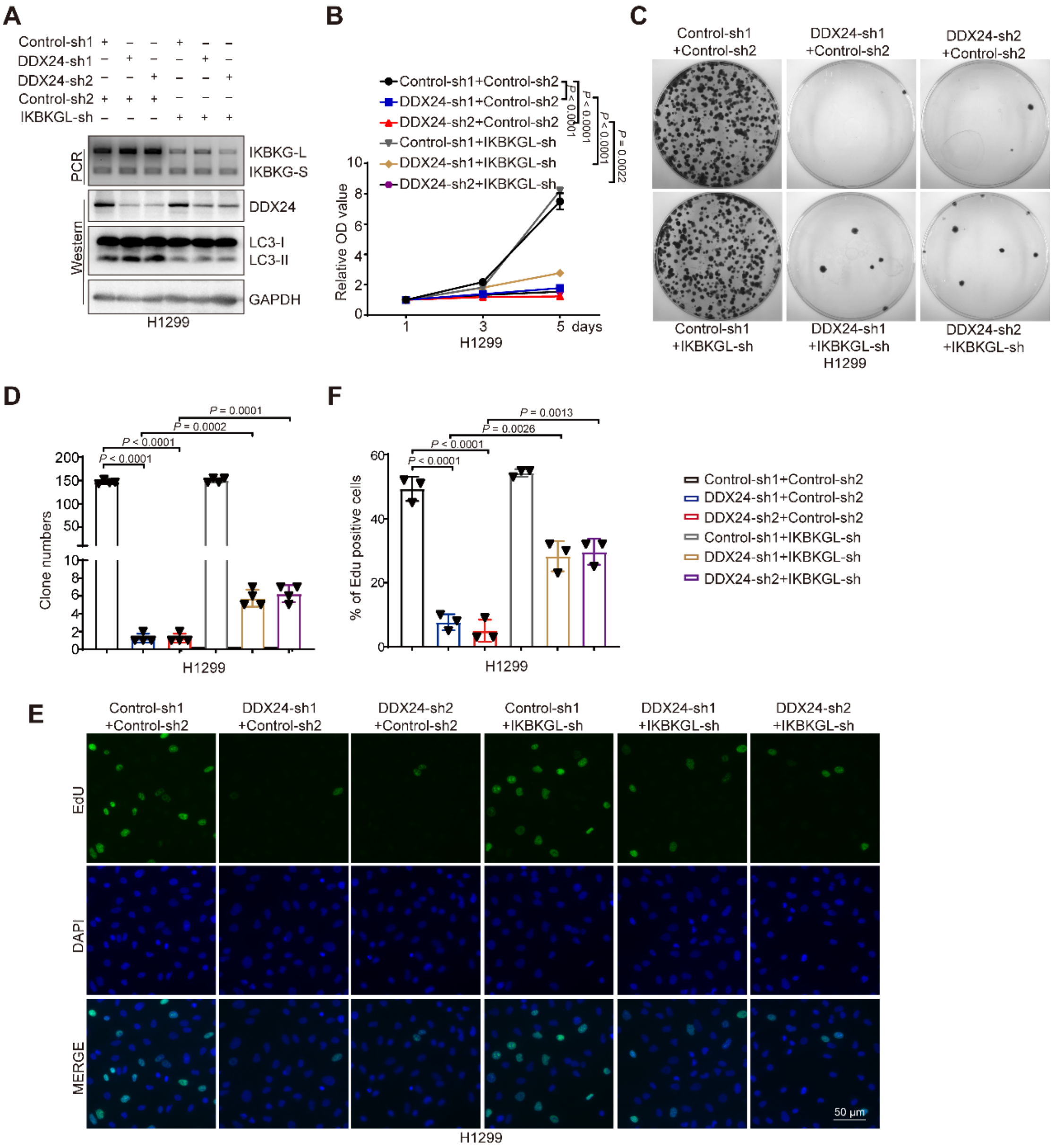

Supplementary Figure 4. Overexpression of IKBKG long isoform restored DDX24-knockdown-induced autophagy and its effect on H1299 cells. (A) The mRNA levels of IKBKGL or IKBKGS were examined by RT-PCR in H1299 cells which stable knockdown DDX24 with or without IKBKGL knockdown. The protein levels of LC3 and DDX24 were examined by western-blots in H1299 cells which stable knockdown DDX24 with or without IKBKGL knockdown. **(B)** CCK8 assays were performed to determine cell growth in H1299 cells which stable knockdown DDX24 with or without IKBKGL knockdown. P values were determined by two-way repeated measures ANOVA. **(C-D)** Colony formation assays using H1299 cells which stable knockdown DDX24 with or without IKBKGL knockdown. Representative pictures of the whole plates from triplicate experiments are shown. The mean ± SD of colony numbers was plotted, with P values calculated by one-way ANOVA with Dunnett' s multiple comparison test, t-test. **(E-F)** The proliferation abilities of H1299 cells which stable knockdown DDX24 with or without IKBKGL knockdown. Quantification

of EdU positive cells were plotted, with P values calculated by one-way ANOVA with Dunnett's multiple comparison test, t-test. Scale bar: 50 μm.
